# Propensity-score-matched evaluation of under-recognition of acute kidney injury and short-term outcomes

**DOI:** 10.1038/s41598-018-33103-9

**Published:** 2018-10-11

**Authors:** Buyun Wu, Li Li, Xiaoyan Cheng, Wenyan Yan, Yun Liu, Changying Xing, Huijuan Mao

**Affiliations:** 1 Department of Nephrology, The First Affiliated Hospital of Nanjing Medical University, Nanjing, 210029 China; 2 Intensive Care Unit, Sichuan Cancer Hospital & Institute, Sichuan Cancer Center, School of Medicine, University of Electronic Science and Technology of China, Chengdu, 610041 China; 3 Department of Information, The First Affiliated Hospital of Nanjing Medical University, Nanjing, 210029 China

## Abstract

Acute kidney injury (AKI) is a common disease, but diagnosis is usually delayed or missed in hospitalized patients. The aim of this study was to investigate the impact of under-recognition of AKI (beyond 3 days after AKI onset) on short-time prognosis. Of 785 patients with under-recognition of AKI and 616 patients with timely-recognition of AKI were propensity matched in a 1:1 ratio. The two groups, with a total of 482 matched patients (241:241), were comparable in baseline covariates. Under-recognition of AKI was not associated with 30-day all-cause mortality in the logistic regression model with covariate adjustment (OR = 1.01, 95% CI = 0.62–1.64, *p* = 0.967). Sensitivity analyses and subgroup analyses also proved the association. There were also no significant differences in causes of 30-day mortality, in-hospital mortality, recovery of renal function at discharge, length of hospital stay, length of intensive care unit stay or hospitalization costs between the two groups, although timely-recognition group had more chance of renal consult and a little more interventions for AKI. In conclusion, under-recognition of AKI may not be associated with poor short-term outcomes of adult hospitalized patients via these propensity-score-matched analyses.

## Introduction

Acute kidney injury (AKI) is an important healthcare burden worldwide and affects about 13.3 million people annually, 85% of whom live in developing countries^1^. AKI is thought to indirectly lead to 1.7 million deaths each year by greatly increasing the long-term risks of the onset and progression of chronic kidney disease (CKD), cardiovascular and cerebrovascular events, and even sepsis^2–6^.

Although AKI is a relatively common disease, timely recognition is not necessarily associated with an optimistic prognosis because fewer than 50% of cases are diagnosed in the early stage of disease^7^. A multi-center survey in China showed that only about 21.2% of AKI cases are timely diagnosed within 3 days^8^. The reasons for delayed diagnosis of AKI include not fully understanding the importance of the non-specific symptoms of the disease and the lack of early and proactive monitoring of renal function of high-risk patients^9^.

Delayed diagnosis of AKI is known to increase the risk of adverse outcomes. For example, Wilson *et al*.^7^ found that delayed diagnosis of AKI was associated with an increase in 30-day mortality by logistic regression analysis with stepwise correction. However, in that study, the correction factors included the largest Sequential Organ Failure Assessment (SOFA) score over the entire course of the disease, which itself could be affected by an actual delay in diagnosis^10^. Thus, the conclusions drawn from a logistic regression model may be somewhat misleading. Although a randomized controlled trial would be the best study design to detect the effects of delayed diagnosis, or under-recognition of AKI on short-term outcomes, such a study is not possible because of obvious violations of ethical standards. Therefore, at present, there actually exists no strong evidence to support the notion that the delayed or missed recognition of AKI would increase the risk of short-term adverse outcomes^11^. Here, a propensity score (PS) matched study was performed to investigate this issue.

## Materials and Methods

### Patients

Patients who were hospitalized in the First Affiliated Hospital of Nanjing Medical University from October 2013 to September 2014 were screened^12^ and those who met the Kidney Disease: Improving Global Outcomes (KDIGO) AKI diagnostic criteria were included in the study. Patients with the following characteristics were excluded: age <18 years, baseline serum creatinine (SCr) <40 μmol/L, stage 5 CKD, or discharged from the hospital within 24 h. The Ethics Committee of Jiangsu Province Hospital approved the protocol of this retrospective observational study (2016-SR-234) and waived the requirement for written consent because analysis conducted anonymously.

### Data collection

The following data were collected from the electronic medical record system and medical documentation in the hospital: demographic data (sex and age), main diagnosis, comorbidities (cardiovascular diseases, diabetes, CKD, malignant neoplasms, liver diseases, and pulmonary diseases), Charlson comorbidity index (CCI)^13^, estimated glomerular filtration rate (eGFR), department in which AKI occurred [Department of Nephrology, other Internal Medicine, Surgery, or Intensive Care Unit (ICU)], Acute Physiology and Chronic Health Evaluation II (APACHE II) score^14^, SOFA score^15^, hospital stay, hospital costs, duration in ICU, one-week pre-AKI drugs used [including contrast agents, chemotherapeutic drugs, angiotensin-converting enzyme inhibitors (ACEIs) or angiotensin receptor blockers (ARBs), diuretics and nephrotoxic antibiotics], risk factors for AKI, AKI stage, use of renal replacement therapy (RRT), interventions after AKI, 30-day mortality, in-hospital mortality, and recovery of renal function at discharge.

### Definitions

The baseline SCr value was set based on the lowest measurement (≥40 μmol/L) during hospitalization^16^. AKI was defined according to the KDIGO criteria as an increase in SCr to ≥26.5 μmol/L within 48 h or ≥1.5 times the baseline value either known or presumed to have occurred within the prior 7 days^17^.

Complete recovery of renal function was defined as the recovery of SCr to no more than the baseline value of 44.2 μmol/L and partial recovery was defined as greater than the baseline value of 44.2 μmol/L, but no more than the maximum value and discontinuing dialysis >1 week at the time of discharge. Failure to recovery was defined as continuous SCr elevation or requirement of maintenance dialysis^18^.

Timely recognition of AKI was defined as diagnosis within 3 days after onset, as documented in the electronic medical record system^8^. Under-recognition of AKI was defined as either a delayed or missed diagnosis. Delayed diagnosis of AKI was defined as recording AKI more than 4 days after onset^8^. Missed diagnosis of AKI was defined as no relevant diagnostic record in the medical record system. In this study, sepsis was defined as infection-induced systemic inflammatory response syndrome according to the 1992 definition^19^. Surgery included elective surgery, time-limited surgery, emergency surgery, and other surgical treatments.

### Outcomes evaluation

The primary clinical endpoint was 30-day all-cause mortality. Secondary clinical outcomes included in-hospital mortality, recovery of renal function at discharge, average length of hospital stay, average length of ICU stay, and hospitalization costs.

### Statistical methods

All data analyses were conducted using SAS version 9.2 statistical software. Continuous variables with a normal distribution were presented as the mean (±standard deviation) and compared between groups using the Student’s t-test. Continuous variables with a skewed distribution were presented as the median (range) and compared between groups using the rank-sum test. Categorical variables were expressed as the number (percentage) and compared between groups using the chi-squared test.

A multivariate logistic regression model was used to yield a PS for each patient. The dependent variable was whether AKI recognition was timely or not. There were 17 independent variables, including sex, age, department that AKI occurred, CCI, eGFR on admission, oliguria, history of CKD, history of malignant neoplasms, APACHE II score, SOFA score, risk factors for AKI (hypovolemia, heart failure, sepsis, surgery), AKI stage, blood urea nitrogen, and whether RRT was received. The PS was used to produce a 1:1 match, which was the closest with no replacement. The caliper was 0.2 times the root mean square value of the standard deviation of the two groups (0.05 after calculation)^20^. Model discrimination was assessed with c statistics (0.913), and model calibration was assessed with Hosmer-Lemeshow statistics (χ^2^ = 12.150; df = 8; *p* = 0.145).

After all PS matches were performed, differences in patient characteristics between the 2 groups were compared by standardized differences, of which 0.8, 0.5, and 0.2 were considered large, medium, and small differences, respectively, and ≥0.1 was defined as meaningful imbalance^21,22^. Survival curves were generated using the Kaplan–Meier method and compared using the log-rank test. To reduce the influence of possible confounding variables, we used the PS matched analysis, inverse probability of treatment weighting (IPTW) and stratification based on quintiles of the PS scores. In the PS-matched cohort, the risks of mortality between groups were compared with logistic regression using Generalized Estimating Equations. A two-sided *p* value of less than 0.05 was considered statistically significant.

## Results

### Baseline characteristics of patients

**Figure 1:**
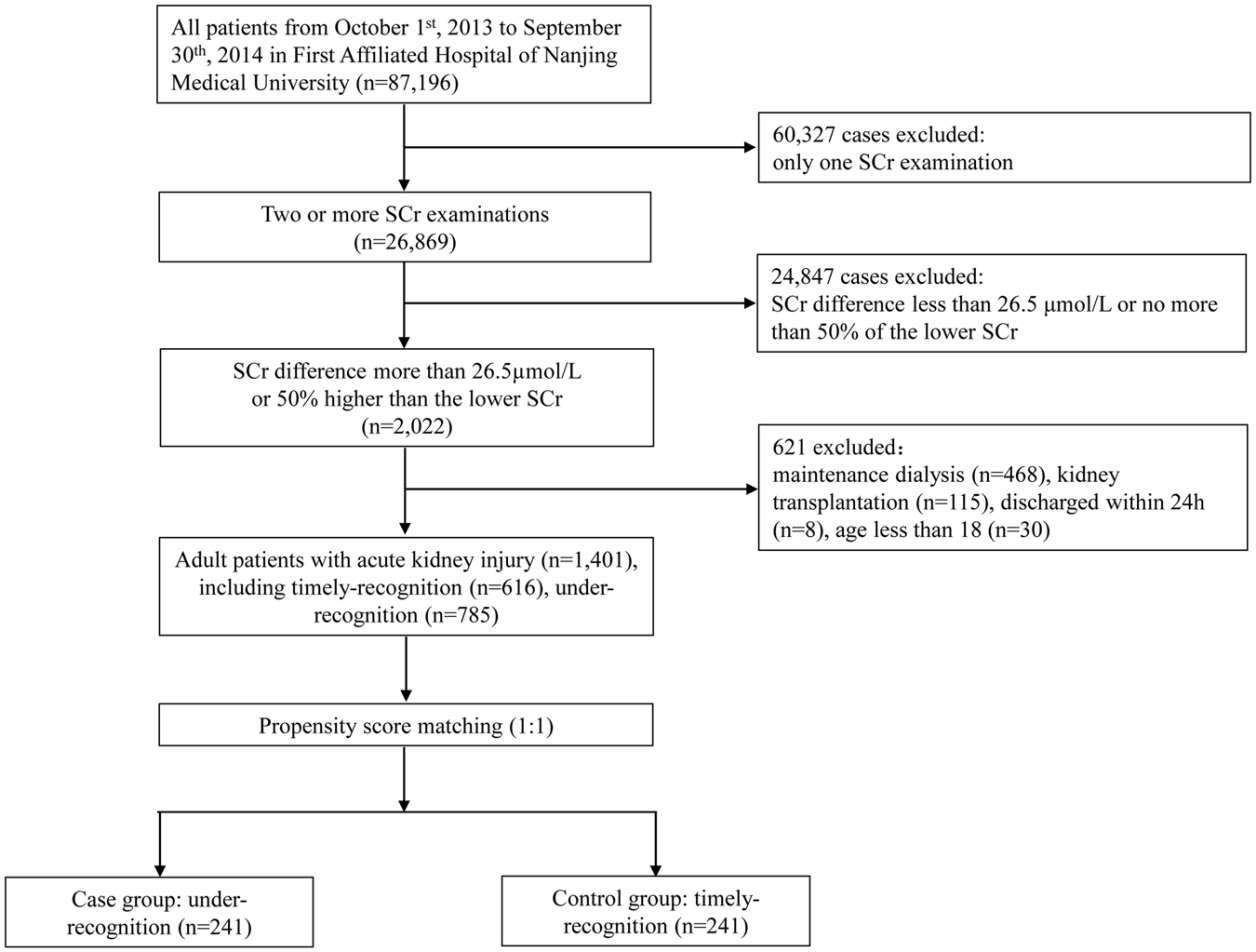
Flowchart of patient selection.

From October 1, 2013 to September 30, 2014, a total of 87,196 patients were admitted to the first Affiliated Hospital of Nanjing Medical University. Of these patients, 26,869 (30.8%) had two or more SCr values during hospitalization and 621 were excluded: 468 with end-stage renal disease, 115 with kidney transplant, 30 aged <18 years, and eight who were hospitalized for <24 h. Finally, 1401 cases (1.61%) in this cohort were diagnosed with AKI (Fig. 1).

According to whether AKI was timely recognized, the patients were divided into two groups: a timely recognition (TR) group (616 cases) and an under-recognition (UR) group (785 cases). There were significant differences between groups in patient age, department distribution, complications (lung disease, malignancy, and CKD), CCI, AKI risk factors (usage of contrast agent, chemotherapeutic agent, heart failure, sepsis, surgery), AKI stage, and laboratory parameters (hemoglobin, platelet, albumin, total bilirubin, urea nitrogen, and eGFR) (Table 1).

**Table 1:** Baseline characteristics of AKI patients in the original and matched cohort.

	Original cohort			Matched cohort		
	Timely-recognition (n = 616)	Under-recognition (n = 785)	Standard differences	Timely-recognition (n = 241)	Under-recognition (n = 241)	Standard differences
**Matched variables**						
Male (%)	415(67.4)	524(66.8)	0.01	167(69.3)	167(69.3)	0.00
Mean age	65.1 ± 17.9	61.7 ± 16.4	0.20	65.3 ± 17.5	66.7 ± 15.8	0.08
Department distribution (%)						
Internet medicine	331(53.8)	292(37.2)	0.33	135(56.0)	129(53.5)	0.05
Surgery	92(14.9)	330(42.0)	0.63	45(18.7)	42(17.4)	0.03
ICU	193(31.3)	163(20.8)	0.24	61(25.3)	70(29.0)	0.08
Malignant tumors	113(18.3)	241(30.7)	0.27	57(23.6)	62(25.7)	0.02
Chronic Kidney disease	85(13.8)	31(4.0)	0.34	23(9.5)	22(9.1)	0.01
CCI	2(1,4)	2(0,3)	0.50	2(1,4)	2(1,4)	0.05
APACHE II score	15(10,26)	11(8,17)	0.50	13(9,24)	14(10,22)	0.02
SOFA score	7(4,11)	5(2,9)	0.48	6(3,10)	5(3,10)	0.05
Oliguria (%)	256(41.6)	44(5.6)	0.93	45(18.7)	36(14.9)	0.09
AKI stage (%)						
1	110(17.8)	422(53.7)	0.80	87(36.1)	85(35.3)	0.02
2	76(12.2)	233(29.7)	0.43	61(25.3)	64(26.5)	0.03
3	430(69.8)	130(16.6)	1.27	93(38.6)	92(38.2)	0.01
Receiving RRT (%)	219(35.5)	9(1.1)		17(7.0)	8(3.3)	0.17
Risk factors of AKI (%)						
Heart failure	198(32.1)	213(27.1)	0.10	81(33.6)	78(32.3)	0.02
Hypovolemia	224(36.4)	297(37.9)	0.03	87(36.1)	81(33.6)	0.05
Sepsis	102(16.5)	52(6.6)	0.31	23(9.5)	30(12.4)	0.09
Surgery	119(19.3)	395(50.3)	0.69	68(28.2)	60(24.9)	0.08
Admission eGFR (ml•min^−1^•1.73 m^−2^)	47.4 ± 36.5	82.8 ± 29.6	1.06	62.3 ± 37.9	62.4 ± 30.5	<0.01
Blood urea nitrogen (mmol/L)	16.6(10.5,25.8)	9.4(6.5,13.7)	0.65	13.0(9.3,20.1)	11.7(8.3,18.1)	0.06
**Unmatched variables**						
Reasons of admission						
Cardiovascular	125(20.3)	249(31.7)	0.26	61(25.3)	62(25.7)	0.01
Pulmonary	119(19.3)	66(8.4)	0.32	34(14.1)	36(14.9)	0.02
Gastrointestinal	40(6.5)	82(10.4)	0.14	20(8.3)	31(12.9)	0.15
Hepatopancreatobiliary	84(13.6)	130(16.6)	0.08	30(12.4)	34(14.1)	0.04
Urinary	129(20.9)	68(8.7)	0.35	36(14.9)	25(10.4)	0.14
Hematological	43(7.0)	74(9.4)	0.09	27(11.2)	24(10.0)	0.04
Neurological	31(5.0)	67(8.5)	0.14	13(5.4)	17(7.0)	0.07
Others	45(7.3)	49(6.2)	0.04	20(8.3)	12(5.0)	0.13
Comorbidities (%)						
Cardiovascular disease	364(59.1)	461(58.7)	0.01	147(61.0)	157(65.1)	0.08
Diabetes	124(20.2)	127(16.2)	0.10	45(18.7)	59(24.5)	0.14
Pulmonary	56(9.1)	35(4.4)	0.18	20(8.3)	16(6.7)	0.06
Liver disease	48(7.8)	58(7.4)	0.01	20(8.3)	18(7.5)	0.03
Interval from admission to AKI (days)	3(1,9)	7(2,12)	0.11	5(1,11)	5(1,10)	0.04
Postrenal AKI	43(7.0)	37(4.7)	0.09	12(5.0)	14(5.8)	0.03
Risk factors of AKI (%)						
Use of contrast agents	59(9.6)	147(18.7)	0.26	32(13.3)	34(14.1)	0.02
Chemotherapy	27(4.4)	56(7.1)	0.18	16(6.7)	22(9.1)	0.09
ACEI/ARB	35(5.7)	44(5.6)	0.00	17(7.0)	26(10.8)	0.13
Diuretics use	218(35.3)	233(29.7)	0.12	101(41.9)	76(31.5)	0.21
Laboratory data						
White blood cells (10^9^/L)	10.7(7.0,15.8)	10.7(7.3,15.4)	0.01	10.1(6.5,14.8)	9.7(6.5,15.0)	0.06
Hemoglobin (g/L)	104(85,122)	111(94,128)	0.25	103(86,122)	107(88,128)	0.10
Platelet counts (10^9^/L)	128(75,197)	141(88,202)	0.09	138(78,198)	146(87,202)	0.05
Serum albumin (g/L)	30.9(26.8,36.3)	34.2(29.9,38.6)	0.44	31.4(26.7,36.8)	31.9(27.9,35.8)	0.04
Serum total bilirubin (µmol/L)	11.3(6.3,26.7)	14.3(9.1,26.6)	0.10	11.5(7.1,23.6)	13.3(8.3,18.1)	0.07

ACEI: angiotensin-converting enzyme inhibitors; AKI, acute kidney injury; ARB: angiotensin receptor blockers; CCI, Charlson comorbidity index; eGFR, estimated glomerular filtration rate; ICU, intensive care unit; RRT, renal replacement therapy.

After propensity score matching, 241 cases were assigned to each group (Fig. 1). After matching, an adequate comparability was shown by a decrease to less than 0.2 of the standardized difference between TR group and UR group for all baseline matched covariates (Table 1). Apart from diuretic usage, there were also no significant differences in other unmatched baselines covariates including reasons for admission, comorbidities, risk factors of AKI and laboratory data on admission (i.e., measurements of white blood cells, hemoglobin, platelets, albumin and serum total bilirubin) (Table 1).

### Endpoints

**Figure 2:**
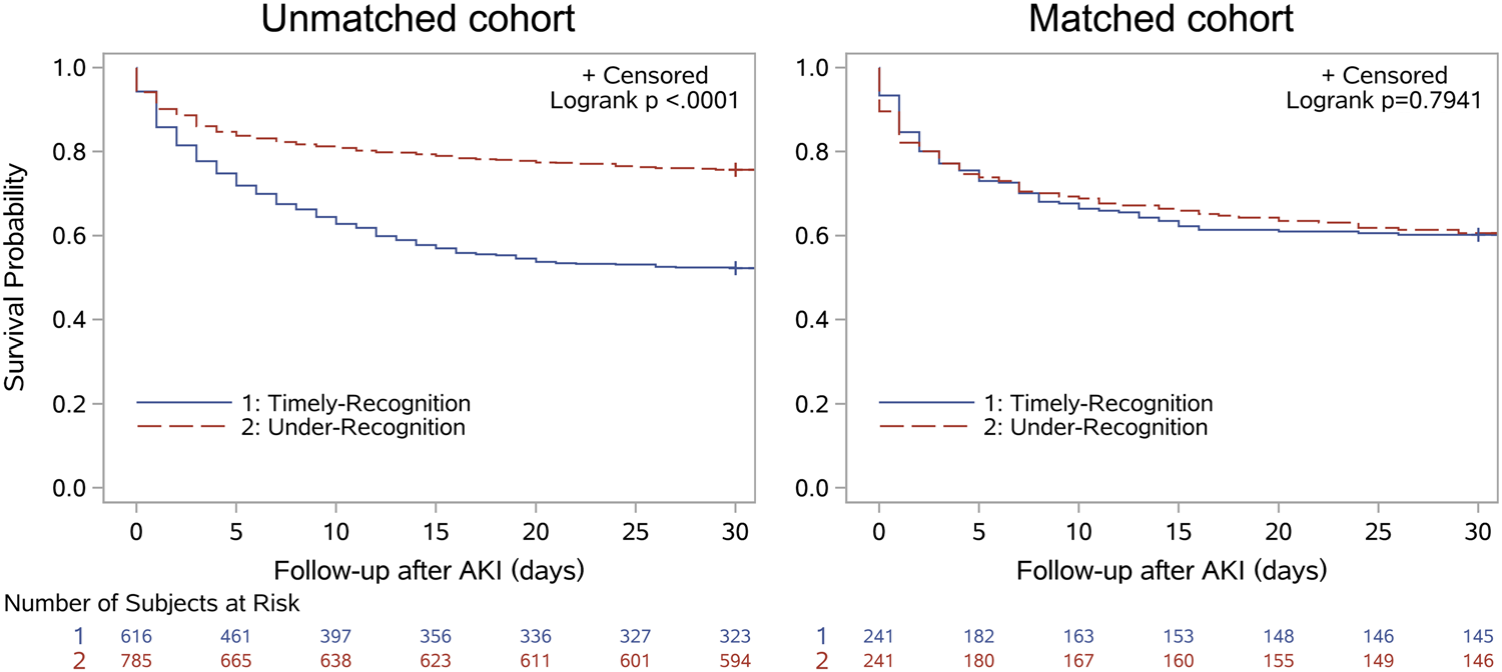
Kaplan–Meier plots of 30-day all-cause mortality after onset of acute kidney injury (AKI).

Within 30 days after AKI, 298 (48.4%) of 616 patients in the TR group and 197 (25.1%) of 785 patients in the UR group had died before matching (*p* < 0.001). After matching, 96 (39.8%) of 241 patients in the TR group and 100 (41.5%) of 241 patients in the UR group had died (*p* = 0.771, Table 2). The Kaplan–Meier plots demonstrated that there was no difference in 30-day mortality between these two groups (*p* = 0.794, Fig. 2). Furthermore, there was no difference in the causes of 30-day mortality (*p* = 0.903, Table S1) or any secondary endpoint (i.e., in-hospital mortality, renal function recovery when discharged, average length of hospital stay, average length of ICU stay, hospitalization costs, and daily hospitalization cost) after matching between the TR and UR groups. The UR group was also not associated with 30-day mortality in univariate and multivariate logistic regression analysis using Generalized Estimating Equations (Tables S2–S3).

**Table 2:** Comparison of endpoints in AKI patients in the original and matched cohorts.

	Unmatched cohort		*p*-value	Matched cohort		*p*-value
	Timely-recognition (n = 616)	under-recognition (n = 785)		Timely-recognition (n = 241)	under-recognition (n = 241)	
**Primary endpoint**						
30-day all-cause mortality (%)	298(48.4)	197(25.1)	<0.001	96(39.8)	100(41.5)	0.711
**Secondary endpoints**						
In-hospital mortality (%)	279(45.3)	180(22.9)	<0.001	94(39.0)	92(38.2)	0.852
Renal recover at discharge (%)			<0.001			0.639
Complete	190(30.8)	539(68.7)		113(46.9)	122(49.8)	
Partial	99(16.1)	104(13.2)		33(13.7)	36(14.9)	
Failure	327(53.1)	142(18.1)		95(39.4)	85(35.3)	
Hospital stays (days)	16(10,26)	19(12,29)	<0.001	17(11,28)	17(10,28)	0.972
Intensive care unit stay (days)	0(0,8)	0(0,3)	0.295	0(0,6)	0(0,6)	0.498
Hospitalization costs (thousand RMB)	46(20,111)	65(30,124)	<0.001	44(18,91)	48(20,101)	0.407
Daily hospitalization costs (thousand RMB)	2.9(1.6,5.8)	3.5(2.1,5.3)	0.067	2.7(1.5,4.8)	3.1(1.6,5.1)	0.258

### Interventions in the AKI patients

Patients in the TR group had greater numbers of renal consults within 3 and 7 days, a higher rate of stopping ACEI/ARB within 7 days following AKI, and higher rate of renal ultrasound examination within 3 days (Table 3). However, there were no differences between the matched groups in stopping nephrotoxic antibiotics within 3 days, use of non-steroidal anti-inflammatory drugs (NSAIDs), use of contrast agents, transfusion within 3 days, urinalysis within 3 days, and SCr measurement within 3 days.

**Table 3:** Interventions after AKI in the matched AKI cohorts.

	Timely-recognition (n = 241)	Under-recognition (n = 241)	*p*-value
Renal consult within 3 days	33(13.7)	14(5.8)	0.008
Renal consult within 7 days	37(15.3)	19(7.9)	0.028
Nephrotoxic antibiotics within 3 days	30(12.4)	30(12.4)	1.000
Stop Nephrotoxic antibiotics within 3 days	22/30	17/30	0.180
Nephrotoxic antibiotics within 7 days	30(12.4)	33(13.7)	0.686
Stop Nephrotoxic antibiotics within 7 days	22/30	22/33	0.568
ACEI or ARB within 3 days	29(12.0)	31(12.9)	0.783
Stop ACEI or ARB within 3 days	15/29	14/31	0.614
ACEI or ARB within 7 days	31(12.9)	34(14.1)	0.689
Stop ACEI or ARB within 7 days	19/31	12/34	0.038
NSAIDS within 3 days	44(18.2)	42(17.4)	0.812
NSAIDs within 7 days	54(22.4)	48(19.9)	0.504
Contrasts within 3 days	2(0.8)	3(1.2)	0.653
Contrasts within 7 days	2(0.8)	7(2.9)	0.093
Transfusion within 3 days	10(4.1)	8(3.3)	0.632
Urinalysis within 3 days	34(14.1)	43(17.8)	0.264
Renal ultrasound examination within 3 days	4(1.6)	0(0)	0.045
SCr tests within 3 days	154(63.9)	139(57.6)	0.162
SCr tests within 7 days	168(69.7)	160(66.4)	0.435

Nephrotoxic antibiotics refer to aminoglycoside or vancomycin. Abbreviations: ACEI, angiotensin-converting enzyme inhibitor; ARB, angiotensin receptor blocker; NSAIDs, non-steroidal anti-inflammatory drugs; SCr, serum creatinine.

### Sensitivity analyses

The association between under-recognition of AKI and 30-day mortality were indicated in Table 4. PS matching using either crude estimate (OR = 1.04, 95% CI = 0.84–1.29, P = 0.711) or covariate adjustment (OR = 1.01, 95% CI = 0.62–1.64, P = 0.967) showed that under-recognition of AKI did not associated with higher 30-day mortality. Due to inherent drawback that reduced sample sizes exist in PS matching, traditional logistic regression, PS as covariate, PS stratification and ITPW were also used to calculate the association in these sensitivity analyses^23^. The univariate logistic regression in overall cohort showed that under-recognition of AKI was associated with reduced 30-day mortality (OR = 0.36, 95% = 0.28–0.45, P < 0.001). However, the association became statistically insignificant (OR = 0.89, 95% = 0.60–1.32, P = 0.575) after adjustment of 17 matched covariates. In addition, the overall estimated treatment effect from stratification based on PS (Table S4), PS as covariate and PS as covariate “doubly robust” also proved this association. In ITPW models, the association became statistically insignificant after adjustment of 17 matched covariates in Table 4.

**Table 4:** Sensitivity analyses: comparison of odds ratio of 30-day mortality from different PS methods and covariate adjustment.

Under-recognition vs. Timely-recognition	Odds Ratio	95%CI	*p*-value
PS Matching^a^ (Crude)	1.04	0.84–1.29	0.711
PS Matching^a^ (covariate adjustment^b^)	1.01	0.62–1.64	0.967
Logistic Regression (Crude)	0.36	0.28–0.45	<0.001
Logistic Regression (covariate adjustment^b^)	0.89	0.60–1.32	0.575
Stratification 5 strata	0.93	0.68–1.28	0.551
PS as covariate	0.92	0.66–1.29	0.626
PS as covariate “doubly robust”^b^	0.90	0.60–1.35	0.602
IPTW	0.72	0.62–0.84	<0.001
IPTW “doubly robust”^b^	0.88	0.72–1.09	0.253

^a^Logistic regression model using Generalized Estimated Equations; ^b^adjusted by 17 matched variables listed in Table 1. IPTW: Inverse Probability of Treatment Weighting; PS: propensity score.

### Subgroup analyses

**Figure 3:**
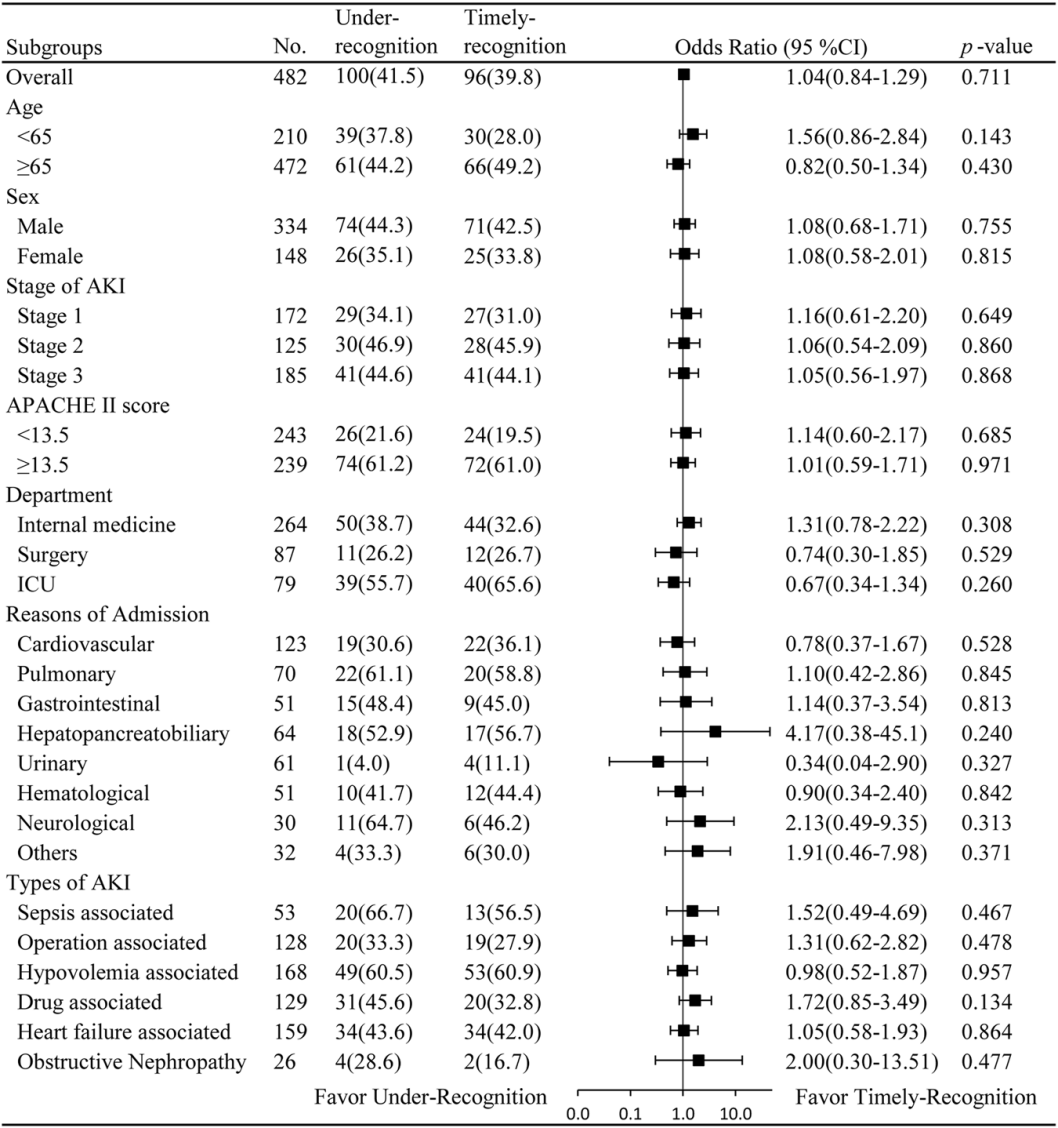
Subgroup analyses of whether under-recognition of AKI increased risk of 30-day all-cause mortality. Abbreviations: AKI, acute kidney injury; APACHE II, Acute Physiology and Chronic Health Evaluation II; ICU: intensive care unit.

Subgroup analyses according to stratification of age, sex, AKI stage, APACHE II score, and department distribution, types of AKI and primary reasons for hospital admission showed that failure to timely recognize AKI did not significantly increase 30-day all-cause mortality in all subgroups in the matching cohort (Fig. 3). In addition, PS matching in specific population with distribution in internal medicine/surgery/ICU department, or with varying types of AKI and primary reasons for hospital admission also proved this result (Table S5).

## Discussion

In this study, the effect of delayed or missed recognition (under-recognition) of AKI on short-term outcomes of adult hospitalized patients was investigated. Using a propensity score matching method, under-recognition of AKI was not associated with a poorer short-term outcome, which was confirmed by the sensitivity analyses and subgroup analyses.

AKI is an important healthcare burden worldwide^24^, but under-recognition of AKI is very common, which may delay the prompt adoption of protective interventions, including volume repletion, blood pressure optimization, hematocrit correction, maintenance of adequate oxygen saturation, and avoidance or discontinuation of nephrotoxic drugs. Therefore, under-recognition of AKI was assumed to increase mortality or limit the recovery of renal function in AKI patients. But, the evidence supporting this viewpoint remains insufficient. In 2008, Wilson *et al*.^7^ found that missed recognition of AKI could increase 30-day mortality by logistic regression stepwise correction, in which the correction factor was the largest SOFA score over the course of the disease. The SOFA score itself could be affected by misdiagnosis of AKI. Thus, this research could not fully confirm the above assumption. Besides, a randomized controlled trial to detect the effect of early recognition of AKI on short-term prognosis is not feasible in the real clinical world. Therefore, this propensity score matching study was designed to preliminarily discuss the issue.

The results of this study showed that under-recognition of AKI did not significantly increase the risk of short-term adverse outcomes, including 30-day all-cause mortality, in-hospital mortality, and recovery of renal function at discharge. There are several possible explanations for these results. First, delayed or missed diagnosis of AKI actually may not be associated with poorer prognosis because of the lack of specific effective treatment for AKI, as prevention may be more important than treatment. A recent randomized controlled study reported that increasing the diagnostic rate of AKI through the use of an electronic warning system did not improve prognosis, which also supported our results^25^. Second, increased recognition rates (such as from 5% to 40%) could lead to improved prognosis by greatly improving the effective treatment of AKI, but when the diagnostic rate increased to a certain level, such as from 40% to 60%, timely diagnosis may lead to little improvement in mortality because available treatment options to further improve prognosis were limited and difficult. Third, timely recognition did not mean very early recognition of AKI, which required physicians to recognize AKI within several hours. Perhaps the use of a more sensitive indicator for very early recognition of AKI could improve outcomes. Lastly, some results may be false-negatives because the median sample size of this study resulted in insufficient power (β = 0.067) to prove these findings. Thus, a larger AKI population is needed to confirm this result.

Early recognition of AKI without adequate intervention did not improve short-term outcomes. Our study showed that the chance of renal consult, the rate of stopping ACEIs or ARBs, the rate of renal ultrasound examination, and the use of diuretics increased in the timely-recognition group as compared to the under-recognition group, although diuretics were not recommended in all cases of AKI with the exception of fluid overload^26^. In addition, our results showed that there were no differences between the matched groups in stopping nephrotoxic antibiotics within 3 days, use of NSAIDs, use of contrast agents, and transfusion within 3 days, which may attribute to the inadequate knowledge and training systems and insufficient multidisciplinary cooperation^27^. Obviously, these factors were not sufficient to improve the short-term outcomes of AKI patients. There was also an increasing trend in the use of RRT in the TR group as compared to the UR group. A recent randomized study showed that an automated, electronic alert system for AKI did not improve outcomes and interventions except frequency of dialysis in surgical patients^25^, which was similar to our results. This indicated that early recognition of AKI without adequate intervention could not improve short-term outcomes. Therefore, adequate intervention is as important as early recognition of AKI.

There were several limitations to this study that should be addressed. First, the sample size of this study (n = 482) resulted in insufficient power (*β* = 0.067). If the difference in the survival rate was 1.7% and α was set to 0.05 and β to 0.90, then 17542 cases would be needed in each group to detect a difference in survival rates. Thus, future studies with larger cohorts are required to verify this hypothesis. Second, the study population was distributed among different departments, so the primary disease, aggravating factors, and risk factors were heterogeneous. The matching method in this study may not be perfect despite the inclusion of 17 factors. Third, short-term, rather than long-term, prognosis was observed in this study because even stage 1 AKI could increase the long-term risk of CKD, and AKI patients with delayed or missed recognition are more likely to develop advanced CKD if there is no long-term follow-up of renal function. Hence, future research of the beneficial effect of improving AKI recognition on long-term outcomes is required.

## Conclusion

The results of this propensity score matched study showed that under-recognition of AKI may not be associated with adverse short-term outcomes in hospitalized adult patients, which may attribute to the inadequate interventions for AKI. Due to the limitation of the sample size, further studies are required to confirm this result. Future studies are also warranted to investigate the effect of under-recognition of AKI on long-term outcomes.

## Electronic supplementary material

Supplementary data

## Data Availability

The datasets generated and/or analysed during the current study are available from the corresponding author on reasonable request.
