# Supplementary material for: Propensity-score-matched evaluation of under-recognition of acute kidney injury and short-term outcomes
Source: Sci Rep. 2018 Oct 11;8:15171. doi: 10.1038/s41598-018-33103-9 (PMC6181969; doi:10.1038/s41598-018-33103-9)
Supplement: Supplementary file 1 — Supplementary data [file 41598_2018_33103_MOESM1_ESM.docx]

**Supplementary data**

**Propensity-score-matched evaluation of under-recognition of acute kidney injury and short-term outcomes**

Buyun Wu, Li Li, Xiaoyan Cheng, Wenyan Yan, Yun Liu, Changying Xing, Huijuan Mao

**Figure S1** Distribution of propensity score before and after matching


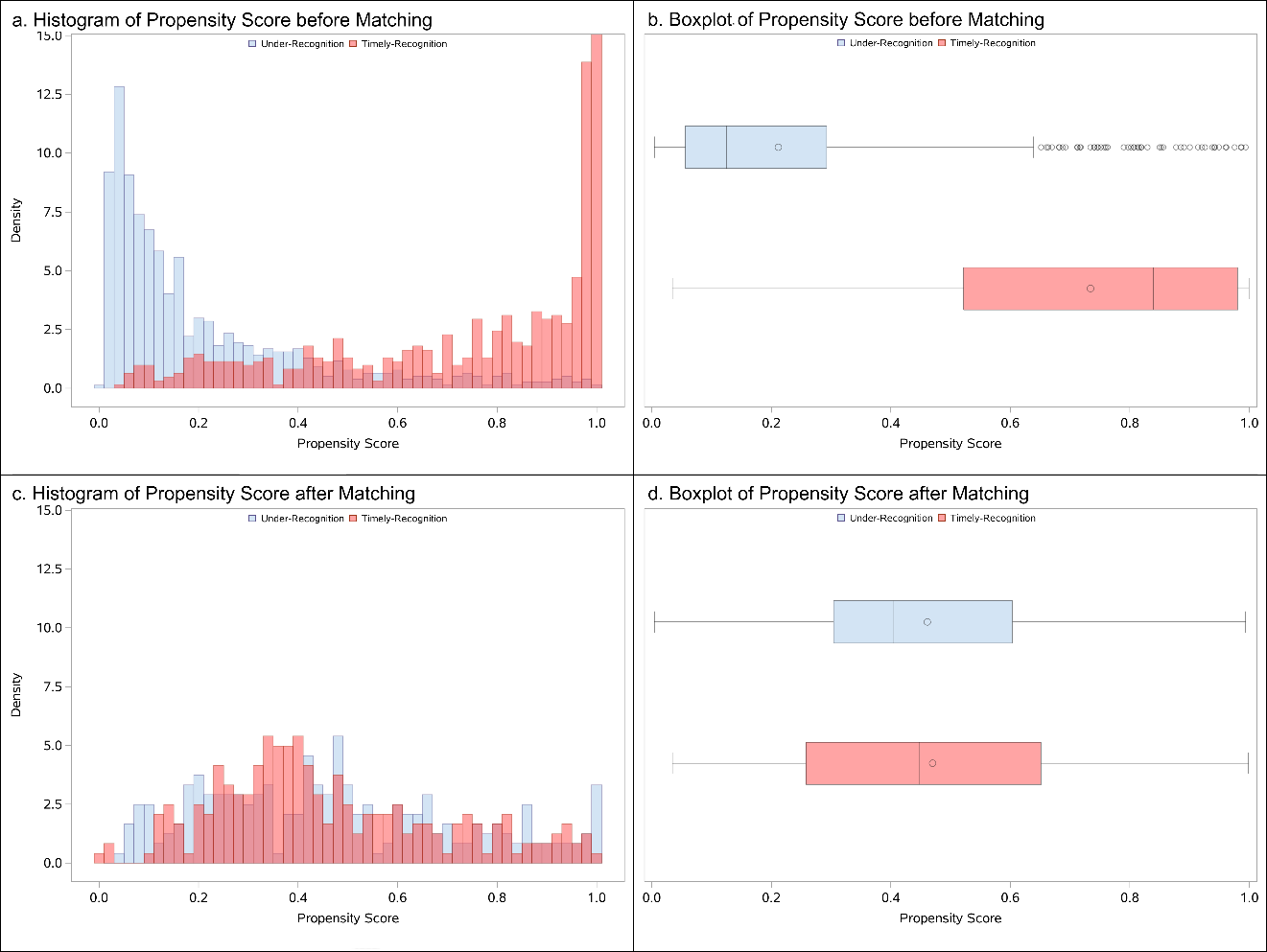


Comments: There were many extreme propensity scores, poor overlap of treatment and control before matching.

**Table S1** Analysis of causes of 30-day mortality in AKI patients in the original and the matched cohorts

|  | Unmatched cohort | |  | Matched cohort | |  | |
| --- | --- | --- | --- | --- | --- | --- | --- |
|  | Timely-recognition  (n=616) | Under-recognition (n=785) | *p*-value | Timely-recognition (n=241) | Under-recognition (n=241) | | *p*-value |
| Number of deaths | 298 | 197 |  | 96 | 100 | |  |
| Causes of death |  |  | <0.001 |  |  | | 0.903 |
| Cardiac disease | 49(16.4) | 25(16.7) |  | 17(17.7) | 15(15.0) | |  |
| Malignant tumors | 82(27.5) | 85(43.1) |  | 38(39.6) | 40(40.0) | |  |
| Pulmonary disease | 13(4.4) | 8(4.1) |  | 4(4.2) | 5(5.0) | |  |
| Pneumonia | 34(11.4) | 10(5.1) |  | 5(5.2) | 9(9.0) | |  |
| Septicemia | 23(7.7) | 7(3.6) |  | 5(5.2) | 4(4.0) | |  |
| Cerebrovascular disease | 17(5.7) | 19(9.6) |  | 6(6.2) | 8(8.0) | |  |
| Kidney disease | 10(3.4) | 0(0) |  | 0(0) | 0(0) | |  |
| Liver disease | 22(7.4) | 10(5.0) |  | 6(6.2) | 6(6.0) | |  |
| Diabetes | 8(2.7) | 0(0) |  | 1(1.0) | 0(0) | |  |
| Suicide | 8(2.7) | 1(0.5) |  | 1(0) | 0(0) | |  |
| Trauma | 2(0.7) | 13(6.6) |  | 1(1.0) | 3(3.0) | |  |
| Aortic dissection | 3(1.0) | 4(2.0) |  | 1(1.0) | 2(2.0) | |  |
| Other and unknown causes | 27(9.1) | 15(7.6) |  | 11(11.5) | 8(8.0) | |  |

.

**Table S2** Univariate logistic regression analyses using Generalized Estimating Equations on risk factors of 30-day all-cause mortality in the matched cohort

| Variables | Univariate logistic regression | |
| --- | --- | --- |
|  | Odds Ratio (95% CI) | *p*-value |
| Sex (male: female) | 1.53(1.01-2.30) | 0.043 |
| Age (per 1-year increment) | 1.02(1.01-1.03) | <0.001 |
| With cardiovascular disease | 0.80(0.55-1.17) | 0.254 |
| With diabetes | 0.78(0.50-1.22) | 0.275 |
| With pulmonary disease | 1.89(0.93-3.86) | 0.079 |
| With liver disease | 1.21(0.65-2.25) | 0.554 |
| With chronic kidney disease | 0.40(0.19-0.82) | 0.012 |
| With malignant tumors | 1.52(0.97-2.38) | 0.067 |
| Charlson comorbidity score | 1.19(1.09-1.29) | <0.001 |
| APACHE II score | 1.12(1.10-1.15) | <0.001 |
| SOFA score | 1.27(1.20-1.34) | <0.001 |
| Hemoglobin (g/L) | 1.00(0.99-1.01) | 0.817 |
| Platelet count (per 10*10^9^/L) | 0.96(0.94-0.99) | 0.002 |
| Serum albumin (g/L) | 0.97(0.94-0.99) | 0.023 |
| Serum total bilirubin when AKI (per 10µmol/L increment) | 1.07(1.04-1.10) | <0.001 |
| Blood urea nitrogen when AKI (per 1mmol/L increment) | 1.00(0.99-1.01) | 0.869 |
| eGFR (per 10 ml•min^-1^•1.73m^-2^ increment) on admission | 1.09(1.03-1.15) | 0.001 |
| Stage 2: 1 of AKI | 1.72(1.07-2.77) | 0.026 |
| Stage 3: 1 of AKI | 1.65(1.08-2.51) | 0.021 |
| Oliguria | 2.98(1.85-4.81) | <0.001 |
| Receiving RRT | 0.51(0.25-1.04) | 0.065 |
| Hypovolemia | 3.63(2.47-5.33) | <0.001 |
| Heart failure | 1.11(0.75-1.64) | 0.590 |
| Surgery | 0.57(0.38-0.87) | 0.009 |
| Sepsis | 2.72(1.52-4.88) | <0.001 |
| Use of contrast agents | 0.84(0.50-1.42) | 0.525 |
| Diuretics usage | 1.40(0.96-2.03) | 0.077 |
| Delayed or missed diagnosis of AKI | 1.07(0.73-1.57) | 0.725 |

AKI: acute kidney injury; APACHE II: Acute Physiology and Chronic Health Evaluation II; eGFR, estimated glomerular filtration rate; RRT, Renal replacement therapy; SOFA: Sequential Organ Failure Assessment.

**Table S3** Multivariate logistic regression analysis using Generalized Estimating Equations on risk factors of 30-day all-cause mortality in the matched cohort

| Variables | Multivariate logistic regression | |
| --- | --- | --- |
|  | Odds Ratio (95% CI) | *p*-value |
| Age (per 1-year increment) | 1.03(1.01-1.04) | 0.004 |
| Charlson comorbidity score | 1.13(1.01-1.28) | 0.037 |
| APACHE II score | 1.06(1.02-1.09) | <0.001 |
| SOFA score | 1.15(1.04-1.26) | 0.005 |
| Serum total bilirubin when AKI (per 10µmol/L increment) | 1.05(1.02-1.09) | <0.001 |
| eGFR (per 1 ml•min^-1^•1.73m^-2^ increment) on admission | 1.08(1.01-1.16) | 0.042 |
| Oliguria | 2.71(1.38-5.20) | 0.004 |
| Receiving RRT | 0.34(0.11-1.00) | 0.049 |
| Surgery | 0.25(0.13-0.48) | <0.001 |
| Delayed or missed diagnosis of AKI | 1.04(0.63-1.72) | 0.869 |

All variables that were found to be potentially statistically significant (*p* < 0.10) in the univariate analysis were included in multivariate analyses, and the table above listed the variables that were found to be statistically significant (*p* <0.05). AKI: acute kidney injury; APACHE II: Acute Physiology and Chronic Health Evaluation II; eGFR, estimated glomerular filtration rate; RRT, Renal replacement therapy; SOFA: Sequential Organ Failure Assessment.

**Table S4** Treatment effect estimate based on the Propensity Score stratification

|  | Group | No. of patients | Mortality (%) | χ^2^ | *p*-value |
| --- | --- | --- | --- | --- | --- |
| Overall | Under-recognition | 785 | 197(25.1) |  |  |
|  | Timely-recognition | 616 | 298(48.4) | 84.87 | <0.001 |
| After stratification into quintiles based on propensity scores^a^ | | | | | |
| Quintile 1 | Under-recognition | 269 | 18(6.69) |  |  |
|  | Timely-recognition | 7 | 0 | 0.00 | 1.000 |
| Quintile 2 | Under-recognition | 241 | 58(24.07) |  |  |
|  | Timely-recognition | 36 | 9(25.00) | 0.01 | 0.903 |
| Quintile 3 | Under-recognition | 175 | 77(44.00) |  |  |
|  | Timely-recognition | 102 | 40(39.22) | -4.78 | 0.686 |
| Quintile 4 | Under-recognition | 72 | 31(43.06) |  |  |
|  | Timely-recognition | 205 | 105(51.22) | 8.16 | 0.598 |
| Quintile 5 | Under-recognition | 14 | 6(42.86) |  |  |
|  | Timely-recognition | 263 | 143(54.37) | 11.51 | 0.563 |
| Treatment effect estimate based on Stratification (Mantel-Haenszel)  Odds Ratio (95% CI) = 0.93 (0.68-1.28) | | | | 0.179 | 0.672 |
| Breslow-Day Test | | | | 3.039 | 0.551 |

There were no difference of treatment effect estimate among the quintiles through the Breslow-Day Test (χ^2^ = 3.039; df = 4; *p* = 0.551). ^a^ seventeen propensity score (1.2%) missed.

**Table S5** Subgroup analyses of whether under-recognition of AKI increased risk of 30-day all-cause mortality in specific population.

| Specific population | Before Matching | | | After Matching | | |
| --- | --- | --- | --- | --- | --- | --- |
|  | No.of UR/TR | Odds Ratio (95% CI) | *p*-value | No.of UR/TR | Odds Ratio (95% CI) | *p*-value |
| Different Departments | | | | | | |
| Internal medicine | 292/331 | 0.72(0.51-1.00) | 0.049 | 121/121 | 1.00(0.59-1.69) | 1.000 |
| Surgery | 330/92 | 0.28(0.15-0.51) | <0.001 | 43/43 | 0.88(0.36-2.14) | 0.782 |
| Intensive care unit | 163/193 | 0.29(0.18-0.45) | <0.001 | 47/47 | 1.00(0.47-2.11) | 1.000 |
| Different types of AKI | | | | | | |
| Sepsis associated | 52/102 | 0.82(0.42-1.60) | 0.555 | 20/20 | 2.45(0.53-11.25) | 0.248 |
| Nephrotoxin associated | 230/122 | 0.33(0.21-0.52) | <0.001 | 52/52 | 1.54(0.64-3.72) | 0.335 |
| Hypovolemia associated | 298/224 | 0.21(0.15-0.31) | <0.001 | 86/86 | 0.87(0.45-1.65) | 0.662 |
| Different reasons of hospital admission | | | | | | |
| Cardiovascular | 249/125 | 0.13(0.08-0.22) | <0.001 | 66/66 | 0.93(0.45-1.95) | 0.858 |
| Pulmonary | 66/119 | 0.37(0.20-0.69) | 0.002 | 23/23 | 1.00(0.28-3.59) | 1.000 |
| Gastrointestinal | 82/40 | 0.56(0.26-1.23) | 0.148 | 17/17 | 0.47(0.13-1.70) | 0.250 |
| Hepatobiliary-pancreatic | 130/84 | 0.24(0.14-0.44) | <0.001 | 28/28 | 0.87(0.32-2.38) | 0.781 |
| Genitourinary | 68/129 | 0.23(0.05-1.04) | 0.056 | 21/21 | 1.00(0.05-18.36) | 1.000 |
| Hematological | 74/43 | 0.59(0.27-1.27) | 0.174 | 14/14 | 0.53(0.09-3.03) | 0.479 |
| Neurological | 67/31 | 0.52(0.22-1.23) | 0.136 | 13/13 | 2.86(0.64-12.67) | 0.167 |
| Others | 49/45 | 0.41(0.17-0.98) | 0.044 | 7/7 | 0.53(0.06-4.43) | 0.560 |

^a^ through univariate logistic regression; ^b^ through logistic regression using Generalized Estimating Equations. AKI: acute kidney injury; TR: timely-recognition; UR: under-recognition.
